# Identification of body size characteristic points based on the Mask R-CNN and correlation with body weight in Ujumqin sheep

**DOI:** 10.3389/fvets.2022.995724

**Published:** 2022-11-02

**Authors:** Qing Qin, Dongliang Dai, Chongyan Zhang, Cun Zhao, Zhichen Liu, Xiaolong Xu, Mingxi Lan, Zhixin Wang, Yanjun Zhang, Rui Su, Ruijun Wang, Zhiying Wang, Yanhong Zhao, Jinquan Li, Zhihong Liu

**Affiliations:** ^1^Key Laboratory of Animal Genetics, Breeding, and Reproduction in Inner Mongolia Autonomous Region, Inner Mongolia Agricultural University, Hohhot, China; ^2^Key Laboratory of Mutton Sheep Genetics and Breeding of Ministry of Agriculture, Inner Mongolia Agricultural University, Hohhot, China; ^3^College of Animal Science, Inner Mongolia Agricultural University, Hohhot, China; ^4^The Inner Mongolia Autonomous Region Goat Genetics and Breeding Engineering Technology Research Center, Inner Mongolia Agricultural University, Hohhot, China

**Keywords:** sheep, regression analysis, weight, body size, machine vision

## Abstract

The measurements of body size data not only reflect the physical fitness, carcass structure, excellent growth condition, and developmental relationship among tissues and organs of animals but are also critical indicators to measure the growth and development of sheep. Computer vision-based body size identification is a non-contact and stress-free method. In this study, we analyzed different body size traits (height at wither, body slanting length, chest depth, chest circumference, shank circumference, hip height, shoulder width, and rump width) and the body weight of 332 Ujumqin sheep and significant correlations (*P* < 0.05) were obtained among all traits in Ujumqin sheep. Except for shoulder width, rump width, and shank circumference, all were positively correlated, and the effect of sex on Ujumqin sheep was highly significant. The main body size indexes affecting the body weight of rams and ewes were obtained through stepwise regression analysis of body size on body weight, in order of chest circumference, body slanting length, rump width, hip height, height at wither, and shoulder width for rams and body slanting length, chest circumference, rump width, hip height, height at wither and shoulder width for ewes. The body slanting length, chest circumference, and hip height of ewes were used to construct prediction equations for the body weight of Ujumqin sheep of different sexes. The model's prediction accuracy was 83.9% for the rams and 79.4% for ewes. Combined with a Mask R-CNN and machine vision methods, recognition models of important body size parameters of Ujumqin sheep were constructed. The prediction errors of body slanting length, height at wither, hip height, and chest circumference were ~5%, chest depth error was 9.63%, and shoulder width, rump width, and shank circumference errors were 14.95, 12.05, and 19.71%, respectively. The results show that the proposed method is effective and has great potential in precision management.

## Introduction

Biometric measurements are used to define various characteristics of animals. Body measurements and live weight are the most commonly used metrics in scientific research and selection applications. Animal body weight and growth characteristics are crucial for stockbreeding; therefore, it is essential to measure these parameters accurately. Research on body measurements is vital for variety identification (1). Weight is an important factor in the production of sheep. Management decisions related to weight are essential for improving the productivity and efficiency of production units. Sheep body size data records not only reflect an animal's body fitness, carcass structure, growth condition, and the relationship between the development of various tissues and organs but are also a key indicator to measure the growth and development of sheep. However, weight and body size measurements are prone to errors, risk of zoonotic disease transmission, and physical contact with sheep, which can cause tremendous stress on sheep and affect their growth and development. These factors become constraints to the development of stockbreeding.

The problem of growth trait determination has been one of the challenges surrounding the development of stockbreeding with the development of machine vision. Using alternative, inexpensive, non-contact machine acquisition methods to replace human observation of animals without disturbing their normal behavior has become a research development direction. Many researchers use image processing to achieve precision in farming populations. Many studies have been conducted in this area in terms of individual livestock tracking, such as facial recognition (2, 3), unique counting (4), trajectory tracking (5, 6), and coat color identification (7); in behavioral determination, such as feeding behavior (8), aggression (9), breeding behavior (10), and gait detection (11); and in performance assessment, such as body condition assessment and weight prediction (12–15).

CNN (convolutional neural network) is one of the representative algorithms of deep learning. It is a feed-forward neural network that includes convolutional computation and has a deep structure. CNN generally consists of a convolutional layer, a pooling layer, a fully connected layer, and an output layer and uses a backpropagation algorithm for the model training process (16). Trained CNNs can extract information from images end-to-end with fast processing speed (17). R-CNN (Region-CNN) is the first algorithm to apply deep learning to target detection successfully. The R-CNN generates about 2,000 candidate regions based on a selective search method. Then resizes each candidate region to a fixed size, feeds it into the CNN model, and feeds the final feature vector into the classifier to predict the probability value of each class of objects contained. The classifier predicts the probability value of belonging to each type of object in the candidate regions (18). However, R-CNN still has a severe speed bottleneck. The reason is also obvious that there is double computation when the computer performs feature extraction for all regions. Faster-R-CNN proposes ROI pooling to solve this problem (19). The Mask R-CNN is a convolutional neural network model proposed by He et al. (20). It is an extension of the Faster R-CNN, which features bounding boxes for objects of interest but also pixel-level segmentation masks. This is called instance segmentation, which requires the correct detection of all objects and precise segmentation of each instance.

Ujumqin sheep is a group of Mongolian sheep found in Inner Mongolia, China (21). About 5 million heads of Ujumqin sheep. It is an excellent meat breed characterized by large body size, fast growth rate, strong meat production performance, and cold and rough feeding resistance. Breeders often use body type traits to implement genetic improvement strategies (22). The fact that Ujumqin sheep are grazing livestock makes an obstacle to weighing and body size measurement, thus making the selection of this breed difficult and slow genetic progress. Studies have shown that neither body weight nor body size alone can explain all genetic variations in size in livestock. A multitrait evaluation, including multiple body lengths and weights, maybe a better option for size selection (23).

We hypothesized that machine measurements would be similar to manual measurements and that there would be a link between body weight and body size traits in Ujumqin sheep. In this study, we use Mask R-CNN convolutional neural network developed as an automated picture acquisition device to automatically calculate the critical body size parameters of Ujumqin sheep. Biostatistical methods were used to analyze the critical body size parameters affecting the body weight of Ujumqin sheep and predict the weight of Ujumqin sheep based on these data, providing managers with an efficient, low-cost method for measuring body size metrics.

## Materials and methods

### Data source

Data for this study were collected from the East Ujumqin Original Breeding Farm, East Ujumqin Banner, Inner Mongolia Autonomous Region, from 120 rams and 230 ewes, for a total of 350 sheep, with animals spanning 1–8 years of age. The data were checked for the complete collection, and the records of 18 sheep were deleted (11 were not recorded, 4 were missing weight data, and three were missing body size data), leaving the measurement data of 332 Ujumqin sheep.

### Data acquisition

When the image data were measured, critical point measurement was unstable due to different body postures and sheep positions. To ensure the smooth image acquisition and the validity of the acquired data, a sheep size and weight data acquisition channel were developed in this study, and a database of the received information was established. The device was designed to facilitate the orderly passage of sheep and ensure the image data collection of a single sheep, preventing the sheep from having a herd effect, which leads to bunching and affects the efficiency and accuracy of data collection.

The image data after the acquisition were saved to the device's controller. The images were uploaded to a high-performance image analysis server via the HTTPS protocol, which reduced the calculation costs of the farm and made it easy to maintain the server in an available location. The analyzed data were saved in the database, and information such as body size and weight was transferred back to the equipment controller for display and downloaded for sheep management.

**Figure 1:**
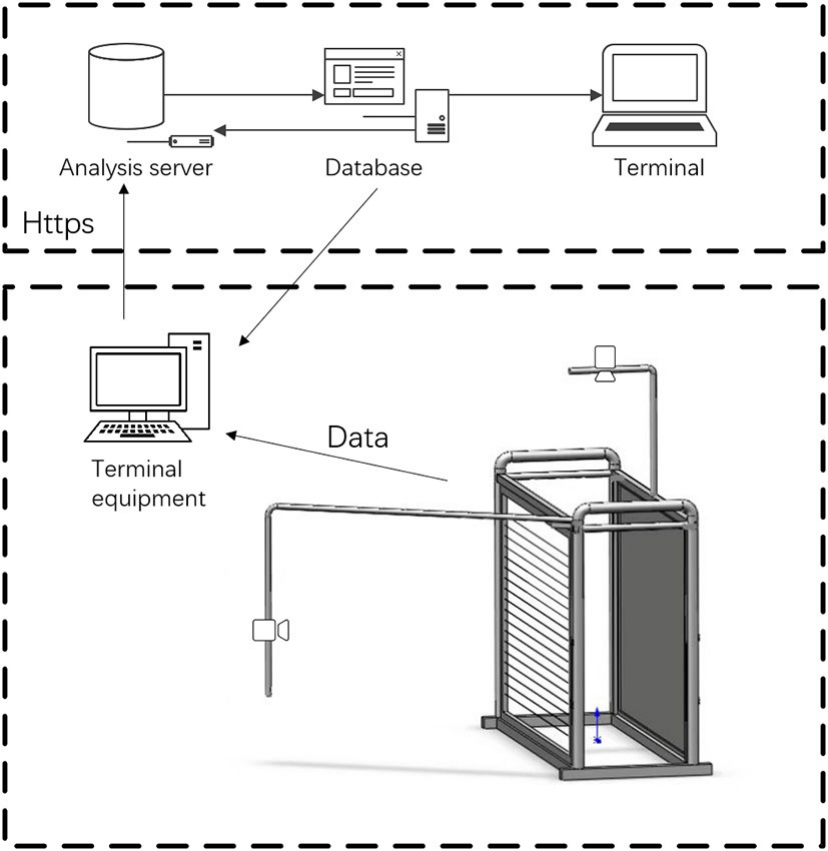
Body size and weight data collection channel. BW, body weight; HW, height at wither; BSL, body slanting length; CD, chest depth; CC, chest circumference; SC, shank circumference; HH, hip height; SW, shoulder width; RW, pump width.

The bottom of the device was integrated with an anti-vibration weight scale for animal husbandry, which can effectively avoid the phenomenon of inaccurate weight measurement due to shaking. The accuracy was 0.5 kg. Two 8 Mega Pixel (MP) autofocus HD camera modules are used on the side and top of the device. The camera model was AX-8M-79 from China. The sensor was a 1/3.2 inch Complementary Metal Oxide Semiconductor (CMOS) IMX179, with a maximum resolution of 3,264 × 2,448, a maximum angle of 65°, and Auto Focus (AF) autofocus. The analysis server's Central Processing Unit (CPU) had 36 cores and 72 threads, with a primary frequency of 2.3 GHz and a core frequency of 3.7 GHz. The memory was Samsung RECC Register Error Checking &Correcting (DDR4) 32 GB, storage is a 2,400 server hard disk, and the graphics card was a TITAN RTX with 24 GB of memory (Figure 1).

### Regression analysis

Body size measurement can accurately reflect the development of the main parts of sheep. Existing tools used for body measurements include measuring sticks, leather rulers, tape measures, and goniometers. Sheep are measured when they are naturally upright and straight on a flat and brightly lit site using calibrating measuring tools. The special staff meter for livestock, the soft ruler, the straightedge, and the collection system was used to determine the body weight and height parameters of the Ujumqin sheep. The body size parameters included body slanting length (BSL), height at wither (HW), chest depth (CD), hip height (HH), shoulder width (SW), rump width (RW), chest circumference (CC), shank circumference (SC), and body weight (BW).

**Figure 2:**
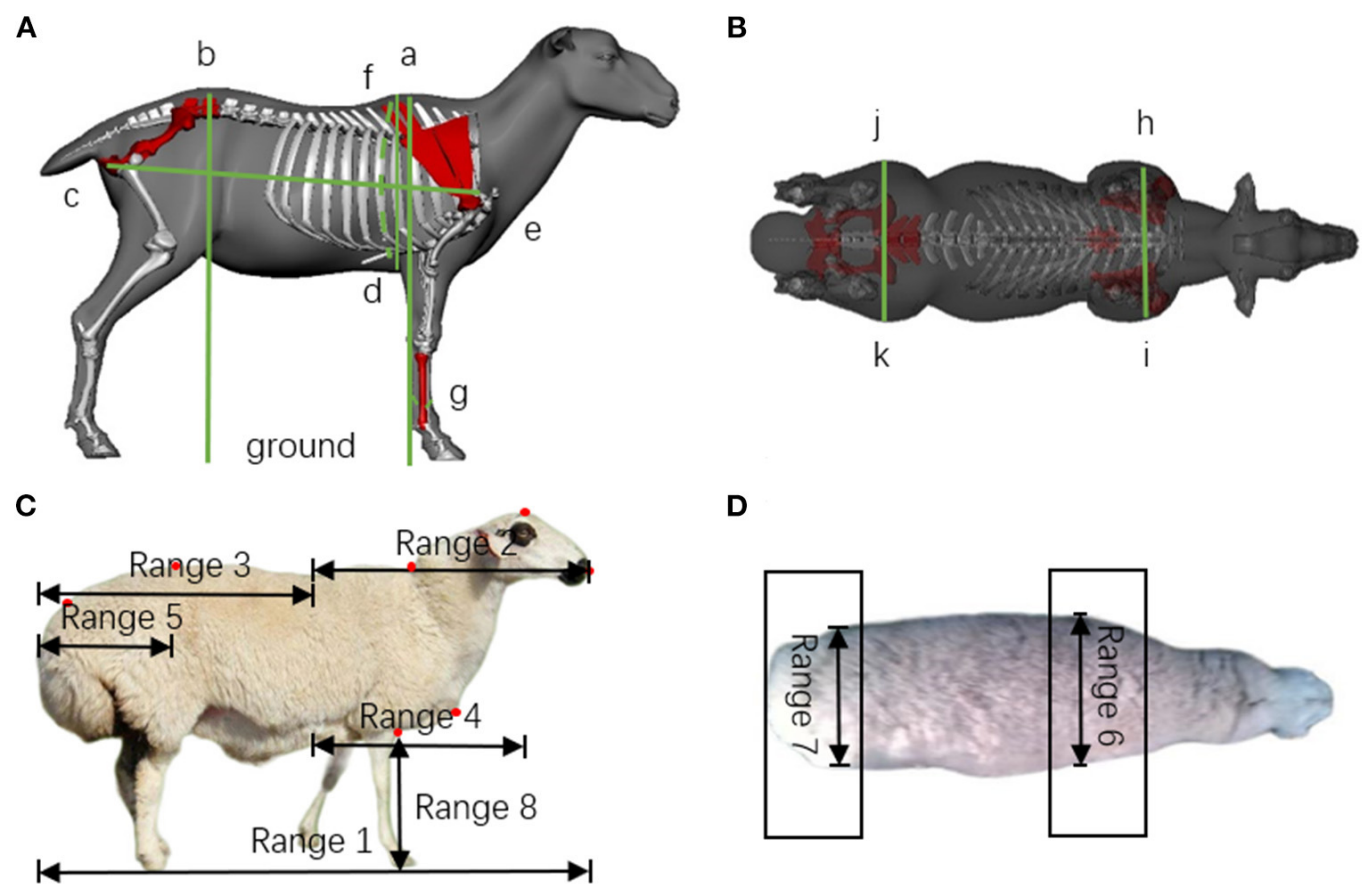
Artificial measurement feature point location diagram and machine visual feature point pickup range. **(A,B)** Show the location of body-scale data measurements on the sheep, HW: a-ground, BSL: c-e, CD: a-d, CC: f-f, SC: g-g, HH: b-ground, SW: h-i, RW: j-k. **(C,D)** Show the range of lateral and dorsal body-scale points measured on the sheep.

1. Height at wither (HW): Vertical distance from the highest point of the shoulder to the ground.
2. Body slanting length (BSL): Distance from the shoulder end's anterior edge to the sciatic tuberosity's posterior border.
3. Chest depth (CD): Straight line distance from the dorsal nail's highest point to the sternum's lower edge.
4. Chest circumference (CC): Vertical circumference of the body torso at the posterior border of the scapula.
5. Shank circumference (SC): Length measured by wrapping a dermatome around the upper 1/3 (thinnest) part of the metacarpal bone of the left forelimb for 1 week.
6. Hip height (HH): Vertical distance from the highest point of the hip to the ground.
7. Shoulder width (SW): Straight line distance between the widest points of the posterior edge of the scapulae on both sides.
8. Rump width (RW): Maximum horizontal width of the outer edge of both hip joints (Figure 2).

In this study, data were collated using Excel 2019 software, and correlation analysis of body size and body weight of Ujumqin sheep was performed using R 4.1.3 and SPSS Statistics 25. Body weight was the dependent variable, and the growth traits mentioned above were introduced into the model one by one as independent variables. See if this variable makes a significant change in the model (*F*-test), and if a significant change occurs, then perform a *t*-test on all variables. When the initially introduced explanatory variables became no longer significant due to the introduction of later explanatory variables, they were removed to ensure that only significant variables were included in the regression equation before introducing a new variable.

### Characteristic point analysis

In this study, 5,000 sheep images were collected in the first stage, of which 70%were used for training sets, 15%were used as verification sets, and the remaining images were used as test sets. The images in the dataset were labeled using CasiaLabeler software, and all images were uniformly tagged with a category. Neural network models were trained. All programs were run in Windows 10 and written in Python based on the Keras deep learning framework.

Radio frequency identification (RFID) sensors were used to read the identification tags, followed by inputting the images of the sides and backs captured by the camera into the image analysis module. The captured images were identified using the Mask R-CNN, which can identify the sheep's location in the image and attach a mask map to the top of the picture. Each image was binarized to obtain the overall contour points of the sheep. The positioning of auxiliary points was also necessary to narrow the range of selected points. The polygon around the outline of the sheep was obtained through the Convexhull function of OpenCV2. Calculate the maximum or minimum value of the specific range of intersecting the contour point to get the edge trajectory of the sheep contour, the auxiliary feature point for the analysis of the body scale algorithm. The body sizes algorithm is as follows.

#### Vertex of the head region of the sheep

The right half of the mask was intercepted, and the range of the convex wrap points is range 2 in Figure 2C. The convex wrap contour points in the right half were sorted, the point with the smallest value on the y-axis was selected, and the point coordinates were returned.

#### Sheep foot point selection

The lower half of the mask was intercepted, and the convex wrap points were picked up in range 2 in Figure 2C. The lower half of the convex wrap contour points were sorted, the point with the largest value on the x-axis was selected, and the point coordinates were returned.

#### Facial vertex

In the normal body position, the rightmost point of the sheep was the top point of the face of the sheep. Therefore, the set of all contour points of the sheep was obtained and sorted, the maximum value on the x-axis was selected, and the coordinates of the point were returned.

The sage nail, the last convexity of the sciatic tuberosity, the anterior edge of the scapula, and the bottom of the thorax were the characteristic points of the profile. In Euclidian space, the curvature of a straight line was zero, and the curvature of an arc was a non-zero constant. According to the principle of maximum curvature, the hip height and body length (sciatic end) measurement points were used to determine the last convexity of the sciatic tuberosity. The anterior sternal edge can also be detected using curvature methods. Still, due to the imprecision of the sternal anterior edge region measurement and the impact of noise, the curvature detection accuracy had a greater effect, so the distance method is used to determine hip height as follows.

The mask's left half was intercepted, and the convex wrap points were picked up in range 3 in Figure 2C. The convex wrap contour points in the left half were sorted, the point with the smallest value on the y-axis was selected, and the coordinates were returned.

#### Body slanting length

First, a straight line was defined through the facial vertex and the sheep's hoof point. The information of all contour points between the facial vertex and the sheep's hoof point was obtained and based on the calculated distance between each of these points, the straight line, the point with the shortest distance was the chest feature point of the body length.


$$\begin{array}{l} d=\frac{\left|ax-by+c\right|}{\sqrt{a^{2}+b^{2}}} \end{array}$$

Where x and y are the point coordinates, ax + by + c is the line equation, and d is the distance from the point to the line.

Next, the maximum curvature point of range 5 in Figure 2C was calculated based on the u-chord length curvature (24), which is the locus at the sciatic tuberosity of the body length. Finally, the contour points of range 5 in Figure 2C are used in the u-chord length curvature formula to calculate the maximum curvature value.

#### Height at wither

Similar to the selection of body slanting length loci, the straight line through the dorsal nail height and the vertex of the head part of the sheep was defined as straight line b, the set information of all contour points between the two points was obtained, and the point with the longest distance was the height at wither feature point according to the formula for calculating the distance between points and lines.

#### Chest depth

The range of the chest depth contour point was determined to be range 4 in Figure 2C, and the maximum curvature point was calculated according to the u-chord length curvature, which is the feature point below the chest depth. The vertical distance of the chest depth was from the bun armor to the sternum. The chest depth was the distance between the y-axis coordinate of the chest depth point and the y-axis coordinate of the bun armor.

#### Shoulder width, rump width

Because of the scapulae and hip joints in sheep, there were protrusions when viewed from the back, limiting the computation area of shoulder width and rump width of Ujumqin sheep. The curvature algorithm was used to calculate the upper and lower loci of the sheep's shoulder width and rump width. The absolute value of the difference in y-values was calculated as the shoulder width and rump width. The shoulder width area was in range 6 in Figure 2D, and the rump width area was in range 7 in Figure 2D.

#### Chest circumference

The cross-section of the thorax of Ujumqin sheep was close to an ellipse. The shoulder width and chest depth can be measured following the dorsal and lateral image measurements, and these two parameters correspond to the long and short axes of the ellipse. The integral formula can be used to find the chest circumference.


$$\begin{array}{l} l=4\int_{0}^{\pi /2}\sqrt{{\left(\alpha cos\alpha \right)}^{2}+\;{\left(\beta sin\beta \right)}^{2}}d\alpha \end{array}$$

Where l is the chest circumference, α is 1/2 the chest width, and β is 1/2 the chest depth.

#### Shank circumference

The cross-section of the shank circumference was close to the circle, and the value range of the contour point is 1/3 of the check point range from Chest Depth to Sheep Foot Point Selection, as shown in Range 8 in Figure 2C. Bring the diameter d of the pipeline to the circle formula *C* = π*d*, and calculate C, which was the shank circumference of the sheep.

The validation set verified the data for traits that needed correction, such as body length, chest circumference, and Shank circumference. A correction coefficient was calculated as the ratio of the two data. Since there was a gap between the camera coordinate system and the actual coordinate system, the pixel distances obtained from the camera measurements were converted into centimeters to get the actual body-scale distance of the sheep more accurately. A test platform was made in this study to control the accuracy of the test height. Considering that the exact dorsal armor height of the Ujumqin sheep does not exceed 100 cm, the range of movement of the back calibration plate was set to 0–105 cm, and the range of motion of the lateral determination plate was set to 0–50 cm, with photographs taken at 5 cm intervals. Since the distance of the calibration plate was known, the pixel conversion relationship corresponding to different lengths was calculated as the various distances. The pixel value/actual centimeter ratio was used as the dependent variable, and the distance was the independent variable. The regression functions for the distances on the back and side were obtained through polynomial regression.

## Results

### Statistical analysis results

The one-way ANOVA showed that body weight, body slanting length, height at wither, chest depth, and chest circumference of Ujumqin sheep were highly significantly influenced by sex. The most significant indexes were body slanting length and height at wither, followed by body weight (*P* < 0.001). Shoulder width, rump width, and shank circumference showed a non-significant effect with sex (*P* > 0.05). The index with the minor effect was shank circumference.

Body weight, body slanting length, height at wither, and hip height data of rams were all higher than those of ewes, indicating that the whole height at wither and length of rams were higher than those of ewes. Shoulder width, rump width, chest depth, and shank circumference were not much different, and the mean chest circumference was higher in ewes than in rams. The coefficient of variation of body weight was the largest, reaching 17.3% for rams and 21.38% for ewes (Table 1).

**Table 1:** Basic data statistics.

	**Sex**	**Mean + SD**	**CV%**	**Max, min**
BW	Male (*n =* 113)^a^	54.09 ± 9.36	17.30%	77, 36
	Female (*n =* 219)^b^	46.06 ± 9.85	21.38%	71, 24.1
	Overall (*n =* 332)	48.83 ± 10.41	21.32%	77, 24.1
BSL	Male (*n =* 113)^a^	72.56 ± 4.4	6.06%	83, 60
	Female (*n =* 219)^b^	67.5 ± 5.14	7.61%	81, 54
	Overall (*n =* 332)	69.22 ± 5.45	7.87%	83, 54
HW	Male (*n =* 113)^a^	69.29 ± 3.85	5.56%	79, 59
	Female (*n =* 219)^b^	63.70 ± 4.15	6.51%	74, 51
	Overall (*n =* 332)	65.63 ± 4.83	7.36%	79, 51
CD	Male (*n =* 113)^a^	30.90 ± 2.68	8.67%	39.5, 25
	Female (*n =* 219)^b^	29.18 ± 4.03	13.83%	39, 19
	Overall (*n =* 332)	29.81 ± 3.75	12.58%	39.5, 19
HH	Male (*n =* 113)^a^	69.84 ± 3.71	5.31%	81, 61
	Female (*n =* 219)^b^	66.33 ± 4.08	6.15%	74, 55
	Overall (*n =* 332)	67.55 ± 4.29	6.35%	81, 55
SW	Male (*n =* 113)^a^	20.61 ± 2.48	12.03%	27, 15
	Female (*n =* 219)^a^	20.21 ± 2.66	13.17%	27, 13
	Overall (*n =* 332)	20.37 ± 2.61	12.81%	27, 13
RW	Male (*n =* 113)^a^	22.74 ± 2.81	12.36%	29, 18
	Female (*n =* 219)^a^	22.12 ± 2.65	11.97%	28, 15
	Overall (*n =* 332)	22.34 ± 2.72	12.18%	29, 15
CC	Male (*n =* 113)^a^	95.88 ± 7.84	8.18%	116, 80
	Female (*n =* 219)^b^	101.31 ± 9.85	9.72%	125, 72
	Overall (*n =* 332)	99.47 ± 9.56	9.61%	125, 72
SC	Male (*n =* 113)^a^	9.04 ± 0.84	9.29%	10.6, 6.5
	Female (*n =* 219)^a^	9.01 ± 0.73	8.08%	11.2, 6.5
	Overall (*n =* 332)	9.03 ± 0.77	8.53%	11.2, 6.5

Different lowercase letters in the same column for the same trait represent significant differences in sex for that shape (P < 0.001), BW, body weight; HW, Wither height; BSL, Body slanting length; CD, Chest depth; CC, Chest circumference; SC, Shank circumference; HH, Hip height; SW, Shoulder width; RW, Rump width.

### Correlation and significance analysis

**Figure 3:**
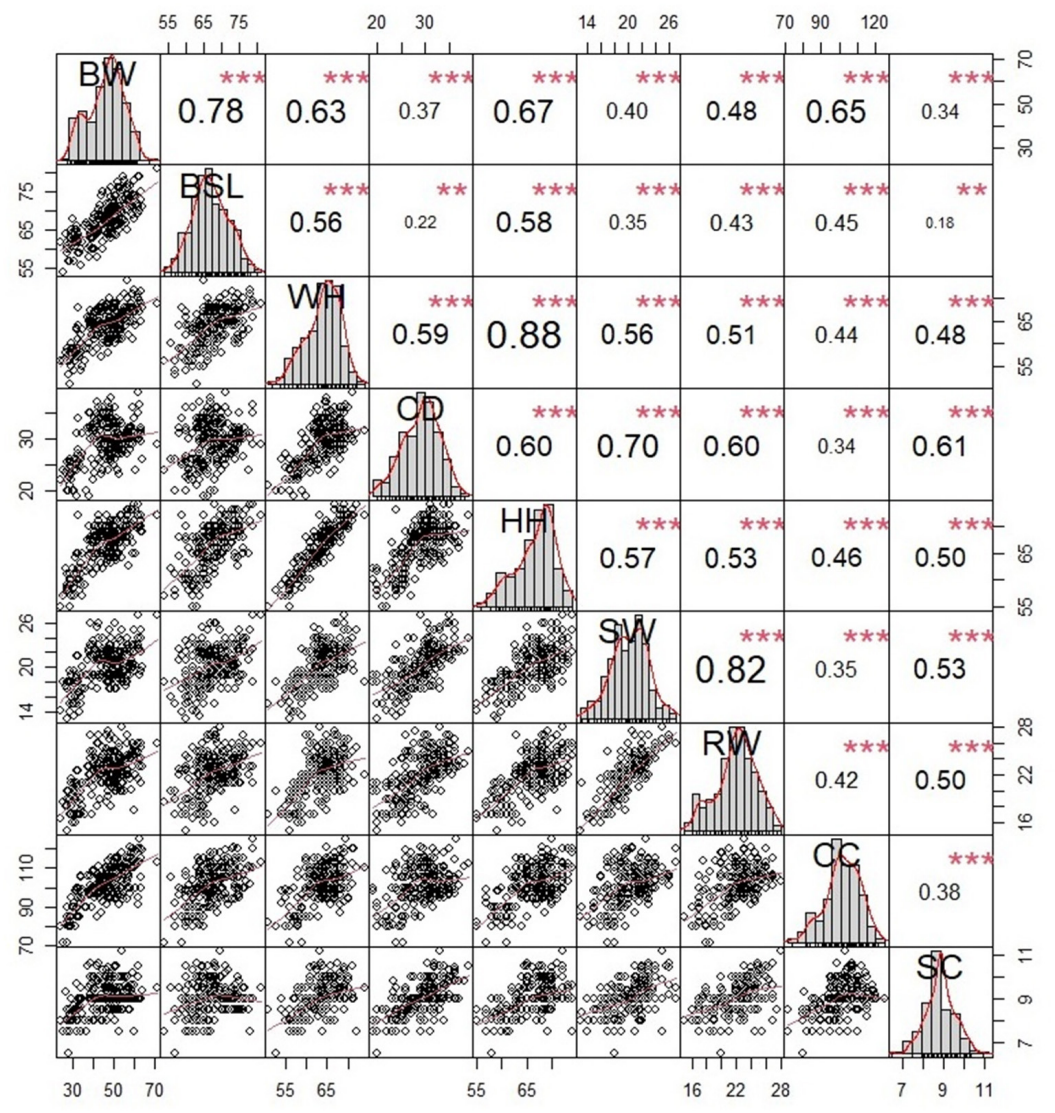
Analysis diagram of the ewe's Spearman correlation. Scatter plots are shown in the lower left corner, correlation-significance plots between traits are shown in the upper right corner, and the data distribution map is shown in the middle. BW, body weight; HW, height at wither; BSL, body slanting length; CD, chest depth; CC, chest circumference; SC, shank circumference; HH, hip height; SW, shoulder width; RW, pump width. *p < 0.05, **p < 0.01, ***p < 0.001.

**Figure 4:**
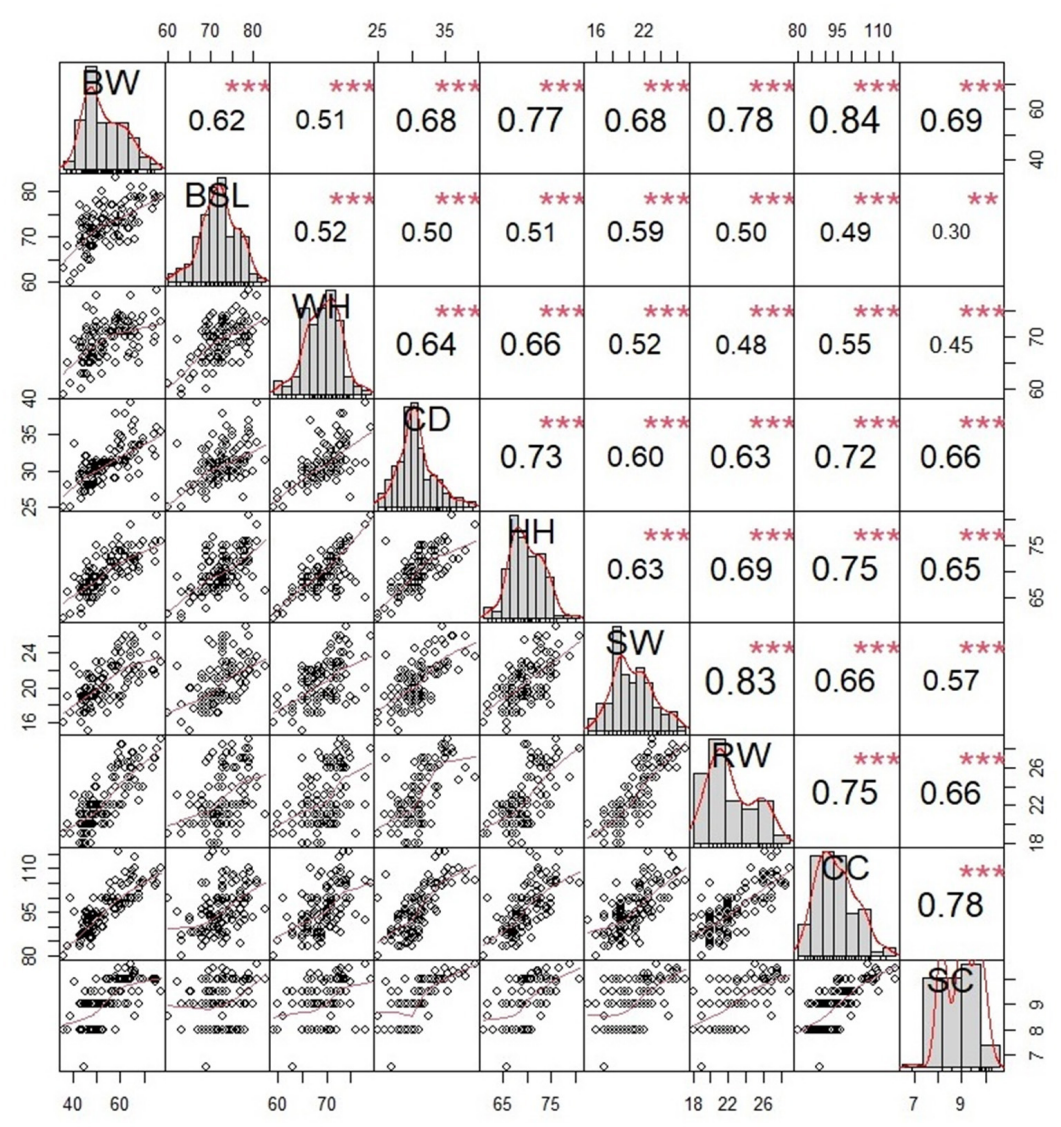
Analysis diagram of the dam's Spearman correlation. Scatter plots are shown in the lower left corner, correlation-significance plots between traits are shown in the upper right corner, and the data distribution map is shown in the middle. BW, body weight; HW, height at wither; BSL, body slanting length; CD, chest depth; CC, chest circumference; SC, shank circumference; HH, hip height; SW, shoulder width; RW, pump width. *p < 0.05, **p < 0.01, ***p < 0.001.

Figures 3, 4 show these traits' Spearman correlation and significance results. It can be concluded that there is a highly significant relationship between body weight and the shape of each foot in Ujumqin sheep (*P* < 0.01). The correlation between body weight and each individual foot in ewes is body slanting length (0.78) >hip height (0.67) >chest circumference (0.65) >height at wither (0.63) >rump width (0.48) >shoulder width (0.40) >chest depth (0.37) >shank circumference (0.34). The correlation between the body weight of rams and each body size in order of size was chest circumference (0.84) >rump width (0.78) >hip height (0.77) >shank circumference (0.69) >shoulder width (0.68) >Chest depth (0.68) >height at wither (0.51). The correlation calculation is the Spearman method, which can better reduce the impact of abnormal values compared to Pearson's test. The ewe height at wither was strongly correlated with hip height (0.88), shoulder width, and rump width (0.82), and the ram weight was strongly correlated with chest circumference (0.84), shoulder width, and rump width (0.83). The ewes showed highly significant correlations between each trait except body slanting length and chest depth (0.22) and body slanting length and shank circumference (0.18). The rams showed highly significant correlations between the traits except for body slanting length and shank circumference (0.30). The scatter plot in the lower left of each figure shows a significant linear relationship between body weight and individual size in Ujumqin sheep. In Figures 3, 4, there is also a positive relationship between the patterns of each body size, indicating that the growth and development of the sheep are whole. As the sheep grew, the weight and various body indicators also followed. In the middle of the data distribution diagram, we can see that the data distribution situation is not fully distributed, so in this study, the Spearman rank relationship is used for correlation analysis, which can avoid the impact of the non-positive distribution of the data.

### Machine vision recognition results

Through the above analysis, the indexes of body size traits that significantly affected the body weight of Ujumqin sheep were chest circumference, body slanting length, hip height, shoulder width, and rump width, where chest circumference requires chest depth and shoulder width for calculation.

**Figure 5:**
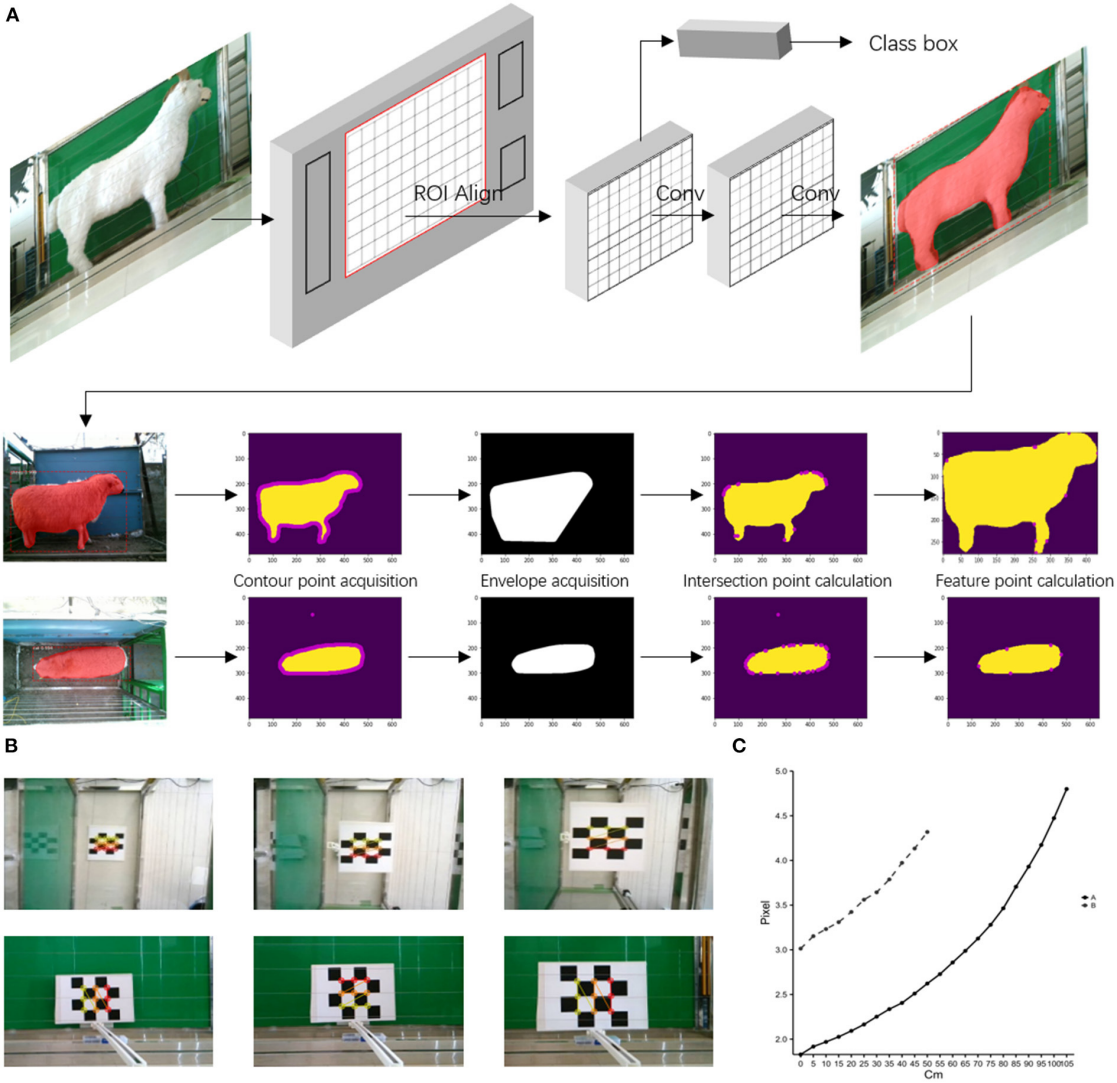
Machine visual recognition flowchart and renderings. **(A)** Shows the flow chart of the Mask R-CNN to recognize the body size of sheep in a natural production environment, **(B)** shows the effect of the calibration plates at different distances, and **(C)** shows the pixel size relationship values per cm from the camera to the calibration plates at different distances, where the dashed line represents the side and the solid line represents the back.

Figure 5A shows the side and back feature point recognition result map. The multistage features of the image are fused by the FPN network, the candidate boxes with high scores are found by calculation and deconvolved, and then the parts are selected in the picture for mask coverage. At this time, the image has completed the first stage of processing. Next, according to the mask map, a binarization process is performed to obtain the contour map of the sheep, and the intersection points of the convex packet map and the contour points are calculated. Finally, the feature point mapping is calculated according to the body size algorithm. Figure 5A shows that the mask coverage of sheep is accurate, and sheep and background can be accurately distinguished. Sheep contour points cover the whole, and the polygon fitted by the convex hull can cover sheep entirely and find the intersection point.

In the above analysis, the pixel distance is obtained by machine calculation, and in associating it with the actual distance, we develop a calibration plate. The size of each calibration plate was 7 × 7 cm, calculated using ∑ai/n, where i is the corresponding pixel value of the picture between two calibration blocks, and n is the number of distances between two adjacent points that can be recognized. From Figure 5B, which shows several ceiling effects at different distances, it can be seen that the camera is accurately calibrated to the corner points of the calibration blocks, and the calibration is good. A regression model is established by constructing the object distance height and centimeter pixel scale relationship. The fitting equation for the back is y1 = 0.0002 × 12 + 0.0015 × 1 + 1.9325. The equation for the side is y2 = 0.0015 × 22 + 0.1008 × 2 + 21.314, where x1 is the height of the calibration plate to the ground, y1 is the back scale relationship, and x2 is the calibration plate to the *R*^2^ of both equations, which is >0.99, indicating that the equation has a high degree of fit (Figure 5).

Among the manually measured body size traits, shoulder width had the most significant coefficient of variation of 11.03%, and height at wither had the smallest coefficient of variation of 4.72%. Among the machine-identified results, the coefficient of variation was the highest for shank circumference (19.71%) and the smallest for body slanting length (4.51%). The relative percentage errors for shank circumference, shoulder width, rump width, and chest depth were large, 19.71, 14.95, 12.05, and 9.63%, respectively. The errors between the manual and machine methods for the other body size traits were ~5%. Overall, this comparison of manual measurements and visual recognition was reliable and relatively stable. The effectiveness of specific image acquisition needs further improvement in future studies (Table 2).


$$\begin{array}{l} Perr=\frac{\left|Da-Dm\right|}{Da}\;*\;100\%\\ \;A=\frac{1}{n}\overset{n}{\underset{i}{\sum }}Perr \end{array}$$

Where Da is the manually measured body size data, Dm is the machine-recognized body size data, and n is the total number of data obtained.

**Table 2:** Percentage error table for manual and machine comparison.

		**BSL**	**HW**	**CD**	**HH**	**SW**	**RW**	**CC**	**SC**	**BW**
Artificial	Mean	71.80	65.45	28.60	67.98	21.18	23.70	105.85	9.03	54.48
	SD	3.88	3.09	2.06	2.84	2.34	1.85	6.67	0.30	7.16
	CV%	5.41%	4.72%	7.21%	4.18%	11.03%	7.79%	6.30%	3.35%	13.14%
Machine	Mean	73.01	64.07	28.91	66.04	23.40	25.35	103.00	9.17	66.42
	SD	5.42	5.57	2.71	4.99	3.07	3.37	7.10	1.81	7.92
	CV%	7.43%	8.69%	9.36%	7.56%	13.14%	13.29%	6.89%	19.71%	11.92%
	A	4.51%	5.65%	9.63%	5.73%	14.95%	12.05%	5.14%	14.94%	24.42%

A is the prediction error of machine and manual measurement. BW, body weight; HW, height at wither; BSL, body slanting length; CD, chest depth; CC, chest circumference; SC, shank circumference; HH, hip height; SW, shoulder width; RW, pump width.

### Stepwise regression analysis

We will measure the body size data with SPSS 25.0 statistical analysis software to gradually return to analysis and establish a diversified linear regression equation of weight and body size.

All data were analyzed by stepwise regression using SPSS 25.0 statistical analysis software to establish a multiple linear regression equation of body weight and body size. Use the F inspection to test the significance of the regression equation, and perform the *T*-test of each interpretation variable that has been selected. When the initially introduced explanatory variables became no longer significant due to the introduction of later explanatory variables, they were removed.

The adult body weight of Ujumqin sheep was predicted using the interdependent morphological characteristics and their principal components. Traits with F < =0.05 were progressively added to the regression analysis with body weight according to the magnitude of the effect. The stepwise regression analysis based on ewes showed that body slanting length, body slanting length and chest circumference, and body slanting length, chest circumference, and hip height explained 59, 74, and 80% of the variation in body weight, respectively. This result was also evident from the highly significant correlation between body weight and body slanting length and chest circumference, and hip height. Different models based on stepwise regression analysis of rams explained 66, 76, 81, 82, 84, and 85% variation in body weight.

The study yielded a model of optimal body weight vs. body size for ewes as BW = −92.49 + 0.82BSL + 0.33CC + 0.75HH (*R*^2^ = 0.797) and for rams as BW = −76.96 + 0.44CC + 0.70BSL + 1.29RW + 0/76HH – 0.46 HW – 0.64SW (*R*^2^ = 0.849) (Table 3).

**Table 3:** Regression equations for different indicators.

	**Component**	**Prediction equation**	**SE**	***R* ^2^**	**Adjusted *R*^2^**	**MSE**	***P*-value**
Ewes	BSL	BW = −53.71 + 1.48BSL	6.35	0.589	0.587	40.357	< 0.001
	BSL and CC	BW = – 70.75 + 1.05BSL + 0.45CC	5.03	0.744	0.742	25.276	< 0.001
	BSL, CC, and HH	BW = – 92.49 + 0.82BSL + 0.33CC + 0.75HH	4.49	0.797	0.794	20.173	< 0.001
Rams	CC	BW = – 39.21 + 0.97CC	5.48	0.664	0.661	29.985	< 0.001
	CC and BSL	BW = – 75.74 + 0.79CC + 0.75BSL	4.62	0.763	0.759	21.318	< 0.001
	CC, BSL, and RW	BW = – 65.74 + 0.51CC + 0.63BSL + 1.13RW	4.19	0.807	0.801	17.556	< 0.001
	CC, BSL, RW, and HH	BW = – 79.42 + 0.41CC + 0.52BSL + 0.96RW + 0.49HH	4.05	0.821	0.815	16.377	< 0.001
	CC, BSL, RW, HH, and HW	BW = – 74.30 + 0.44CC + 0.66BSL + 0.88RW + 0.78HH – 0.52SW	3.83	0.842	0.834	14.648	< 0.001
	CC, BSL, RW, HH, HW, and SW	BW = – 76.96 + 0.44CC + 0.70BSL + 1.29RW + 0/76HH – 0.46 HW – 0.64SW	3.75	0.849	0.841	14.087	< 0.001

BW, body weight; HW, height at wither; BSL, body slanting length; CD, chest depth; CC, chest circumference; SC, shank circumference; HH, hip height; SW, shoulder width; RW, pump width.

## Discussion

In this study, we automatically calculated the body size parameters of sheep using the Mask R-CNN convolutional neural network and analyzed key body size parameters affecting the body weight of Ujumqin sheep, Using biostatistical methods and constructed weight prediction equations based on significant numbers. The traits of Ujumqin sheep showed significant correlations (Figures 3, 4), and all were positively correlated. Chest circumference had the highest correlation with body weight in ewes, and body slanting length had the highest correlation in rams, followed by chest circumference. This finding is the same as that of other researchers (25, 26). It indicates that chest circumference and body length are significant indicators of sheep's weight traits. According to the significance analysis, shank circumference was significantly correlated with all features except body slanting length. It has been suggested in the literature that breed selection can be based on shank circumference, and the larger the shank circumference is, the larger the body size of the livestock. More economic benefits can be produced (27). In the stepwise regression analysis of Ujumqin sheep, the impact of adding shank circumference on prediction weight was not significant, and the effect of gender sex on the sizes of shank circumference was not significant. Therefore, managers should focus on chest circumference and body slanting length data during production to judge the growth of sheep.

Based on the results, it is clear that the effect of sex on the body weight of Ujumqin sheep is very significant (Figure 2), which is consistent with the objective pattern. This result is consistent with previous studies (24, 28). Sex significantly affected all body size traits in Ujumqin sheep, except shoulder width, rump width, and shank circumference. In this study, the body weight, body slanting length, height at wither, and hip height data of rams were all higher than those of ewes, indicating that the whole height at wither and length of rams were higher than those of ewes. Shoulder width, rump width, chest depth, and shank circumference were not much different, and the mean chest circumference was higher in ewes than in rams. Therefore, the sex factor needs to be considered when breeders are screening livestock.

There is a complex correlation between body size and body weight, and researchers have conducted studies using predictive methods such as linear and non-linear regression, decision trees, and neural networks. The most commonly used approaches are the univariate linear regression approach, polynomial regression, and non-linear functions. These methods have two problems. The first is that the approach requires high accuracy and precision of the data. A slight deviation will accumulate, causing large fluctuations in the prediction results and numerous unstable factors on a farm, such as lighting, motion posture, and recognition errors. The result is that this approach is rarely applied to weight prediction using machine vision. Second, compared to multiple linear regression, one-dimensional linear regression and polynomial regression have too few fitted indicators, and the value of individual indicators greatly influences the prediction. Due to the development of machine vision, more body size indicators can be obtained more easily and quickly without worrying about disease transmission and sheep stress. It also allows multiple linear regression for predicting body weight to provide a base data source.

Cominotte et al. used a computer vision system to predict the weight of Nellore cattle, comparing four prediction methods: multiple linear regression (MLR), most minor absolute shrinkage and selection operator (LASSO), partial least squares (PLS) and artificial neural network (ANN). It was shown that the neural network methods' prediction accuracy (RMSEP) ranged from 2.32 to 5.32% for a sample of 234 individuals, with a significant variation in prediction accuracy for different developmental periods of the same model. Machine learning requires substantial computational power, high time cost, and a large sample size, and this study was similar in its sample size. There was no significant difference in prediction accuracy between conducting machine learning and linear regression, so the prediction method of linear regression was used in this study.

The current image segmentation and body size recognition research mainly focus on pose and behavior analysis. The use of machines to carry out body size measurement is especially in standing posture, and algorithms for image segmentation and feature point extraction for body size measurement in the walking state of sheep have not been reported. Menesatti et al. used fixed points in the text for marking, which can deviate when the sheep move and cause problems with the accuracy of the determination (15). Tasdemir et al. used images to manually calibrate feature points. Although animal stress problems were avoided to some extent, the manual steps prevented the integration of such methods into high-throughput applications capable of handling thousands of animals (29). Previously, the point with the most significant curvature on the sheep envelope was the rump measurement point according to the D-P algorithm and the Helen formula. This paper uses the u-chord length curvature method, which has better advantages in terms of rotation resistance and noise immunity. In this study, a measurement channel was established, and a body size recognition model was built according to the skeletal characteristics of sheep, which can automatically select points and measure body size data according to the auxiliary points when facing a large sample size. Through the calculation method of body size feature points in this article, the positions of feature points in the figure are compared with the actual measurement position of body size. Recognizing critical parts of sheep body size is obtained with better effect.

Deep learning methods have been successfully applied to many computer vision tasks in recent years, surpassing many traditional image analysis techniques. Machine vision recognition of body size is more used in pigs and cattle and less used in sheep (30). Compared with previous studies, this study constructs an automated recognition algorithm based on convolutional neural networks and adds the recognition of sheep body size feature points for action states. The method does not require pre-segmentation into many steps, such as image processing, feature extraction, and final prediction (31). Figure 5A shows that in actual production, the method of using Mask-RCNN can accurately distinguish sheep and the background. The researchers also proved the feasibility of this method of actual output (32, 33).

As seen from the back-side fitting equation diagram, the fitted curve shows a curved trend, which is in line with the law of image capture of closer is large and farther is small. By fitting the correct conversion relationship between pixels and centimeters, the ratio of pixels to the actual distance has a low computational cost, which can be better applied to an actual production process.

By comparing the data measured by the machine with those measured manually, it shows that in actual production, the indicators of body slanting length, height at wither, hip height, and chest circumference are relatively accurately measured by the machine vision method. The error of chest depth is around 10%, while the shank circumference, shoulder width, and rump width errors are relatively large. Menesatti et al. developed a low-cost dual-network camera high-resolution system that was tested on the Alpagota Goat. Height at wither and chest depth (~3.5%) and body length (~5.0%) were obtained (34). Bai et al. obtained an error of 4.03% for body length using a model sheep measurement (35). The difference may be because sheep were measured in November, the wool was thicker, which affected the mask coverage effect of the Mask R-CNN, and the back was down due to inertia. So the measurements of body slanting length, height at wither, and hip height were relatively accurate, while chest depth, due to the wool in the image, was higher than the actual measurement. The wool affects the shoulder width and rump width of the identification position of the left and right sides. In the manual measurement, the thickness of the wool is ignored, so the shoulder width and rump width measurement errors are significant. Because the wool near the sheep's hooves significantly influences the diameter, the shank circumference error is significant.

This study were designed based on the Ujumqin sheep, on the one hand, because the images of different species are very different. When applied to other species, the model may not perform as expected. Therefore, recollecting images to construct the neural network according to different species and breeds is necessary. On the other hand, in different breeds, the body size feature points are not precisely the same, and some traits require manual measurements to calculate the correction factor of the body size. It is used to balance errors caused by hair and tails. Therefore, the greater the amount of data in the sample, the better the overall effect of the experiment.

In this study, measurement channels were established, and a body size recognition model based on the skeletal characteristics of sheep was developed to automatically select points and measure body size data based on auxiliary points when faced with large sample sizes. It reduces stress in livestock due to stress, fear, discomfort, or pain. Managers can also access data more quickly and efficiently. The risk of zoonotic diseases is reduced. This study provides a highly efficient and cost-effective way for farmers to collect data. After comparing manual and machine identification data and measurement locations, it is clear that this method is more effective in identifying essential parts of sheep's body size.

## Conclusions

In this study, we propose a method to automatically calculate body size parameters (height at wither, body slanting length, chest depth, chest circumference, shank circumference, hip height, shoulder width, and rump width) and construct weight prediction equations by calculating significant body size parameters based on biostatistical methods. The prediction errors of growth traits were found to be in the range of 4.51–19.71% by machine identification, with a minor prediction error for body slanting length and the largest error for shank circumference. The accuracy of the stepwise regression equation for predicting body weight using growth traits was 83.9% for rams and 79.4% for ewes.

This study provides an efficient, low-cost way for farmers to collect data. However, more research is needed to assess whether such associations exist in other species and for additional trait identification methods. We hope that this study's results will improve sheep's welfare.

## Data availability statement

The original contributions presented in the study are included in the article/supplementary material, further inquiries can be directed to the corresponding author/s.

## Ethics statement

This study was approved by the Scientific Research and Academic Ethics Professional Committee of Inner Mongolia Agricultural University, and was responsible for the Ethical Review of Biomedical Research in Inner Mongolia Agricultural University [Approval No. (2020) 056]. These activities do not require special permits and do not involve endangered or protected species. Written informed consent was obtained from the owners for the participation of their animals in this study.

## Author contributions

ZhihL and QQ contributed to the concept and design of the study. QQ, DD, CZhang, and CZhao data analysis. ZhichL, XX, and ML code modification. ZhixW, YZhan, RS, RW, ZhiyW, YZhao, and JL provided technical support. QQ and DD wrote the first draft of the manuscript. All authors participated in revising the manuscript and reading and approving the submitted version.

## Funding

This work was supported by Major Science and Technology Projects of Inner Mongolia Autonomous Region (2020ZD0004), National Key R&D Program of China (2021YFD200901), Key Technology Project of Inner Mongolia Autonomous Region (2020GG0030), and the National Natural Science Foundation of China (32060742).

## Conflict of interest

The authors declare that the research was conducted in the absence of any commercial or financial relationships that could be construed as a potential conflict of interest.

## Publisher's note

All claims expressed in this article are solely those of the authors and do not necessarily represent those of their affiliated organizations, or those of the publisher, the editors and the reviewers. Any product that may be evaluated in this article, or claim that may be made by its manufacturer, is not guaranteed or endorsed by the publisher.

## References

[1] Yilmaz O Cemal I Karaca O. Estimation of mature live weight using some body measurements in Karya sheep. Trop Anim Health Prod. (2013) 45:397–403. 10.1007/s11250-012-0229-7 22829355

[2] Hansen MF Smith ML Smith LN Salter MG Baxter EM Farish M. Towards on-farm pig face recognition using convolutional neural networks. Comput Industry. (2018) 98:145–52. 10.1016/j.compind.2018.02.016

[3] Noor A Zhao Y Koubâa A Wu L Khan R Abdalla FYJC. Automated sheep facial expression classification using deep transfer learning. Comput Electr Agric. (2020) 175:105528. 10.1016/j.compag.2020.105528

[4] Huang W Zhu W Ma C Guo Y Chen C. Identification of group-housed pigs based on gabor and local binary pattern features. Biosyst Eng. (2018) 166:90–100. 10.1016/j.biosystemseng.2017.11.007

[5] Ahrendt P Gregersen T Karstoft H. Development of a real-time computer vision system for tracking loose-housed pigs. Comput Electr Agric. (2011) 76:169–74. 10.1016/j.compag.2011.01.011

[6] Poursaberi A Bahr C Pluk A Berckmans D Veermäe I Kokin E., editors. Online lameness detection in dairy cattle using body movement pattern (Bmp). In: 11th International Conference on Intelligent Systems Design and Applications. Cordoba; London: IEEE Xplore (2011).

[7] Kim HT Ikeda Y Choi H. The identification of Japanese black cattle by their faces. Asian Australas J Anim Sci. (2005) 18:868–72. 10.5713/ajas.2005.86822221020

[8] Cangar Ö Leroy T Guarino M Vranken E Fallon R Lenehan J. Automatic real-time monitoring of locomotion and posture behaviour of pregnant cows prior to calving using online image analysis. Comput Electr Agric. (2008) 64:53–60. 10.1016/j.compag.2008.05.014

[9] Kim J Chung Y Choi Y Sa J Kim H Chung Y. Depth-based detection of standing-pigs in moving noise environments. Sensors. (2017) 17:2757. 10.3390/s17122757 29186060 PMC5751748

[10] Tsai D-M Huang C-Y. A motion and image analysis method for automatic detection of estrus and mating behavior in cattle. Comput Electr Agric. (2014) 104:25–31. 10.1016/j.compag.2014.03.003

[11] Stavrakakis S Li W Guy JH Morgan G Ushaw G Johnson GR. Validity of the microsoft kinect sensor for assessment of normal walking patterns in pigs. Comput Electr Agric. (2015) 117:1–7. 10.1016/j.compag.2015.07.003

[12] Doeschl-Wilson A Green D Fisher A Carroll S Schofield C Whittemore CJMS. The relationship between body dimensions of living pigs and their carcass composition. Meat Sci. (2005) 70:229–40. 10.1016/j.meatsci.2005.01.010 22063479

[13] Kashiha M Bahr C Ott S Moons CP Niewold TA Ödberg FO. Automatic weight estimation of individual pigs using image analysis. Comput Electr Agric. (2014) 107:38–44. 10.1016/j.compag.2014.06.003

[14] Khojastehkey M Aslaminejad AA Shariati MM Dianat R. Body size estimation of new born lambs using image processing and its effect on the genetic gain of a simulated population. J Appl Anim Res. (2016) 44:326–30. 10.1080/09712119.2015.1031789

[15] Menesatti P Costa C Antonucci F Steri R Pallottino F Catillo GJC. A low-cost stereovision system to estimate size and weight of live sheep. Comput Electr Agric. (2014) 103:33–8. 10.1016/j.compag.2014.01.018

[16] LeCun Y Bengio Y Hinton G. Deep learning. Nature. (2015) 521:436–44. 10.1038/nature14539 26017442

[17] Zhang J Zhuang Y Ji H Teng G. Pig weight and body size estimation using a multiple output regression convolutional neural network: a fast and fully automatic method. Sensors. (2021) 21:3218. 10.3390/s21093218 34066410 PMC8124602

[18] Girshick R Donahue J Darrell T Malik J editors. Rich feature hierarchies for accurate object detection semantic segmentation. In: Proceedings of the IEEE Conference on Computer Vision Pattern Recognition. Columbus, OH; London: IEEE Xplore (2014).

[19] Ren S He K Girshick R Sun J. Faster R-Cnn: Towards real-time object detection with region proposal networks. IEEE Trans Pattern Anal Mach Intell. (2017) 39:1137–49. 10.1109/TPAMI.2016.2577031 27295650

[20] He K Gkioxari G Dollár P Girshick R editors. Mask R-Cnn. In: Proceedings of the IEEE International Conference on Computer Vision. Venice; London: IEEE Xplore (2017).

[21] Na RS Zhao QJ Jin DP Su XH Chen XW Guan WJ. Establishment and biological characteristics of ujumqin sheep fibroblast line. Cytotechnology. (2010) 62:43–52. 10.1007/s10616-010-9260-6 20383581 PMC3303005

[22] Jenkins T Kaps M Cundiff L Ferrell CL. Evaluation of between-and within-breed variation in measures of weight-age relationships. J Anim Sci. (1991) 69:3118–28. 10.2527/1991.6983118x 1894547

[23] Hubbard DD. Guidelines for Uniform Beef Improvement Programs. United States Edition. Beltsville, MD: Extension Service (1981). p. 17.

[24] Sowande O Sobola OS. Body measurements of West African dwarf sheep as parameters for estimation of live weight. Trop Anim Health Prod. (2008) 40:433–9. 10.1007/s11250-007-9116-z 18575971

[25] Benyi K. Estimation of liveweight from chest girth in pure and crossbred west African goats. Trop Anim Health Prod. (1997) 29:124–8. 10.1007/BF02632332 9203315

[26] Robles Jimenez LE Ruiz Perez JA Nicolas DL Chay Canul AJ Ramirez-Rivera JC Villegas-Estrada D. Productive behavior in growing kid goats and methane production with the inclusion of chokecherry leaf (*Prunus salicifolia*). Trop Anim Health Prod. (2020) 52:1257–67. 10.1007/s11250-019-02124-5 31728954

[27] Al-Atiyat RM Tabbaa MJ Barakeh FS Awawdeh FT Baghdadi SH. Power of phenotypes in discriminating awassi sheep to pure strains and from other breeds. Trop Anim Health Prod. (2021) 53:139. 10.1007/s11250-021-02578-6 33495970

[28] Lupi T Nogales S León J Barba C Delgado JJA. Characterization of commercial and biological growth curves in the segureña sheep breed. Anim Int J Anim Biosci. (2015) 9:1341–8. 10.1017/S1751731115000567 25903216

[29] Tasdemir S Ozkan IA. Ann approach for estimation of cow weight depending on photogrammetric body dimensions. Int J Eng Geosci. (2019) 4:36–44. 10.26833/ijeg.427531

[30] Wang Z Shadpour S Chan E Rotondo V Wood KM Tulpan D. Asas-Nanp symposium: applications of machine learning for livestock body weight prediction from digital images. J Anim Sci. (2021) 99:skab022. 10.1093/jas/skab022 33626149 PMC7904040

[31] Fernandes AF Dórea JR Valente BD Fitzgerald R Herring W Rosa GJM. Comparison of data analytics strategies in computer vision systems to predict pig body composition traits from 3d images. J Anim Sci. (2020) 98:skaa250. 10.1093/jas/skaa250 32770242 PMC7447136

[32] Qiao Y Truman M Sukkarieh S. Cattle segmentation and contour extraction based on mask R-Cnn for precision livestock farming. Comput Electr Agric. (2019) 165:104958. 10.1016/j.compag.2019.104958

[33] Xiao J Liu G Wang K Si Y. Cow identification in free-stall barns based on an improved mask R-Cnn and an Svm. Comput Electr Agric. (2022) 194:106738. 10.1016/j.compag.2022.106738

[34] Menesatti P Costa C Antonucci F Steri R Pallottino F Catillo GJC. A low-cost stereovision system to estimate size and weight of live sheep. Comput Electr Agric. (2014) 103:33–8. 10.1080/07494467.2014.906702

[35] Bai M Xue H Jiang X Zhou Y editors. Body size measurement of sheep based on machine vision. In: 2nd International Conference on Manufacturing Science and Information Engineering. Zhuhai: Springer (2017).

